# The Behavioral and Immunological Impact of Maternal Separation: A Matter of Timing

**DOI:** 10.3389/fnbeh.2014.00192

**Published:** 2014-05-22

**Authors:** Susana Roque, Ana Raquel Mesquita, Joana A. Palha, Nuno Sousa, Margarida Correia-Neves

**Affiliations:** ^1^Life and Health Sciences Research Institute, School of Health Sciences, University of Minho, Braga, Portugal; ^2^ICVS/3B’s – PT Government Associate Laboratory, Braga, Portugal; ^3^Neuropsychophysiology Laboratory, Center for Research in Psychology (CIPsi), School of Psychology, University of Minho, Braga, Portugal

**Keywords:** maternal separation, depressive-like behavior, CD8^+^ T cells, T cell CD4/CD8 ratio, corticosterone, anxious-like behavior

## Abstract

Maternal separation (MS), an early life stressful event, has been demonstrated to trigger neuropsychiatric disorders later in life, in particular depression. Experiments using rodents subjected to MS protocols have been very informative for the establishment of this association. However, the mechanism by which MS leads to neuropsychiatric disorders is far from being understood. This is probably associated with the multifactorial nature of depression but also with the fact that different research MS protocols have been used (that vary on temporal windows and time of exposure to MS). In the present study, MS was induced in rats in two developmental periods: for 6 h per day for 14 days between postnatal days 2–15 (MS_2–15_) and 7–20 (MS_7–20_). These two periods were defined to differ essentially on the almost complete (MS_2–15_) or partial (MS_7–20_) overlap with the stress hypo-responsive period. Behavioral, immunological, and endocrine parameters, frequently associated with depressive-like behavior, were analyzed in adulthood. Irrespectively from the temporal window, both MS exposure periods led to increased sera corticosterone levels. However, only MS_2–15_ animals displayed depressive and anxious-like behaviors. Moreover, MS_2–15_ was also the only group presenting alterations in the immune system, displaying decreased percentage of CD8^+^ T cells, increased spleen T cell CD4/CD8 ratio, and thymocytes with increased resistance to dexamethasone-induced cell death. A linear regression model performed to predict depressive-like behavior showed that both corticosterone levels and T cell CD4/CD8 ratio explained 37% of the variance observed in depressive-like behavior. Overall, these findings highlight the existence of “critical periods” for early life stressful events to exert programing effects on both central and peripheral systems, which are of relevance for distinct patterns of susceptibility to emotional disorders later in life.

## Introduction

Depression is a devastating and prevalent mental disorder that causes great disability in modern societies and is predicted to rank in the second position for premature death in 2030 (Mathers and Loncar, 2006). The inability to adequately cope with stress has been implicated as an important factor on the onset and exacerbation of depression (Dinan, 2005; Cohen et al., 2007). Stressful events during the first days of life have been shown to impact on adult behavior (Aisa et al., 2007; Garner et al., 2007; Lee et al., 2007; Seckl, 2008; Mesquita et al., 2009) and to increase vulnerability to neuropsychiatric diseases (Nemeroff, 2004).

The impact of early developmental stressors on neuroendocrine homeostasis, particularly on the hypothalamic–pituitary–adrenal (HPA) axis, is well-recognized (Meaney et al., 1989; Liu et al., 1997; Lehmann et al., 2002). Maternal separation (MS), one of the best well-studied developmental disruptors, is documented to interfere with the maturation process of the HPA axis (Clarke, 1993; Plotsky and Meaney, 1993; Slotten et al., 2006) as well as with other physiological systems such as serotoninergic neurotransmission (Mesquita et al., 2007).

Particular relevant for MS-induced depressive-like behavior are studies using the forced swimming test (FST), revealing increased immobility time in animals submitted to MS (3–4 h per day during the first 15 postnatal days, which overlaps most of the stress hypo-responsive period in rodents) when compared with animals reared in typical housing conditions (Ruedi-Bettschen et al., 2005; Lee et al., 2007; Lambas-Senas et al., 2009; Martisova et al., 2012). Similarly, with respect to the anhedonic dimension of depression, measured by the sucrose preference test (SPT), MS animals display decreased sucrose consumption (Michaels and Holtzman, 2007). However, these results have not been confirmed by others, with respect to both immobility time in the FST (Marais et al., 2008) and sucrose consumption in the SPT (Shalev and Kafkafi, 2002). These discrepancies in the literature, although possibly caused by small changes in the MS protocols used (Schmidt et al., 2011), deserve further investigation, specifically on the mechanisms underlying depressive-like behavior.

Alterations in the immune system have been associated with both early stressful life events and depressive-like behavior (Miller, 2010). However, surprisingly, the potential interplay of the immune system and depression has only been scarcely explored in the MS model. So far, studies in non-human primates early deprived from social contact showed that there is a decrease in the ratio of the two most important T lymphocyte populations, the CD4^+^ and the CD8^+^ T cells (CD4/CD8), and a significant increase in the number and activity of natural killer (NK) cells (Lewis et al., 2000). Similarly, a decrease in the percentage of CD4^+^ T cells and an increase in CD8^+^ T cells were detected in children deprived of maternal care (Gogberashvili, 2006). However, no effect on cell number was observed in rodents submitted to MS (Kruschinski et al., 2008). Considering that the function of the immune cells can be inferred by parameters such as the type and quantity of cytokines produced, it is of notice the short- (Dimatelis et al., 2012) and long-term (Avitsur et al., 2013) decrease in cytokines and chemokines production observed in animals submitted to MS.

To further understand the interplay between the HPA axis and several parameters of the immune system and their relationship with depressive-like behavior, we made use of MS protocols. The duration of the MS period used in this study (6 h/day), previously used by our team and others (Matthews and Robbins, 2003; Colorado et al., 2006; Mesquita et al., 2007), was selected to induce a significant disruption in the mother–pup interaction. Moreover, to identify critical developmental periods relevant for the emergence of these phenotypes, we applied the MS protocol in two neurodevelopmental time windows. One that overlaps most of the stress hypo-responsive period in rats [that usually occurs between postnatal day 4–14 (Schmidt et al., 2011)], and another in which the MS starts after the first postnatal week, where some maturation of the HPA axis has already occurred.

## Materials and Methods

### Animals and maternal separation protocol

The results presented in the study are originated from two independent experiments. Wistar rats (Charles River, Barcelona, Spain) were used in the experiments and maintained under standard laboratory conditions with artificial 12 h light/dark cycle: lights on from 8:00 a.m. to 8:00 p.m., ambient temperature of 22 °C, and 55% of relative humidity; food and water were available *ad libitum*. The mating procedure was the same for all females. A male was introduced in the female’s cage where two virgin females were housed, at the beginning of the dark cycle. Vaginal plug was examined at the beginning of the light cycle. When the presence of the vaginal plug was observed the female was individually housed until delivery. In each experiment, 9–12 females primiparous rats were used. Nest material was provided to each dam and no bedding changes were performed in the last days of pregnancy. The day on which a female rat showed a vaginal plug was designated as embryonic day 0 and the day of delivery as postnatal day 0. Litters were delivered by spontaneous partum on gestation day 22. Pups from all litters were mixed on the day of delivery, and randomly assigned to each dam; the size of each litter was adjusted to 8 (*n* = 4 male and *n* = 4 female, whenever possible). Each dam and the corresponding litter were randomly assigned to one of the following experimental groups (three to five litters for each group): (a) MS from the 2^nd^ to the 15^th^ postnatal day (MS_2–15_); (b) MS from the 7^th^ to the 20^th^ postnatal day (MS_7–20_); and (c) control group with no MS (Cont).

In each experiment, pups from the MS groups were daily separated from their mothers between 9 a.m. and 3 p.m. as previously described (Mesquita et al., 2007); each litter was placed together in a new cage, inside an incubator at 33–35 °C in order to maintain constantly the body temperature of the pups, as previously described (Diehl et al., 2012; Cao et al., 2013). After the 6 h of separation, each MS litter returned to their home cages, where the dam remained. Pups from control litters were left undisturbed with their dams until the weaning day (P21). In the present study, only males, pair-housed at weaning, were analyzed. All experiments were conducted in accordance with National and European regulations (European Union Directive 86/609/EEC) and were approved by the National Veterinary Directorate and by the local Animal Ethical Committee.

### Behavioral tests

At 3 months of age, behavior was evaluated in the open-field (OF) followed by the FST with a 1-day interval. The animals performed both behavioral tests.

In the OF, the animals were individually tested during 5 min in an arena formed by a white square base (43.2 cm × 43.2 cm) surrounded with acrylic transparent walls (ENV – 515; MedAssociates, VT, USA). Illumination was provided by a white bright light. The session started with the animal placed in the center of the arena and, using a system of 16 evenly spaced infra-red sourced and sensors juxtaposed around the periphery of the four sides of the chamber (at 2.5 cm height) with the help of the tracking software that detects solely the movement of the center of the animal’s body (SOF-811, Med Associates, VT, USA). These sensors were connected to a computer, which allowed the following parameters to be recorded: (a) time spent in the central area of the arena (10.8 cm × 10.8 cm; a measure of anxious-like behavior); (b) total distance traveled (a measure of general locomotor activity); and (c) number and duration of rears, manually recorded by two experimenters independently (a measure of exploratory activity).

The FST was chosen to assess behavioral despair, a measure of depressive-like behavior. For this test animals were placed in transparent acrylic cylinders with 40 cm of diameter filled with water (25 °C) to a depth (50 cm depth) such that the animals had no solid support for their rear paws. The test lasted for 5 min and was preceded, 24 h before, by a 10 min pre-test session. At the end of each session, animals were dried and placed under a heating lamp for 15 min before returning to their home cages. The cylinders were filled with fresh water after each trial. A video camera was used to record test sessions from a top angle; video recordings were later scored by an investigator blind to the experimental details. Time of immobility and latency to immobility were computed.

### Corticosterone determination

Blood samples were collected at sacrifice between 10 and 12 a.m.; the interval between transferring animals from their undisturbed environment to decapitation was kept under 60 s. Serum corticosterone levels were assessed by radioimmunoassay, using ImmuChemTM Corticosterone-125I kits (MP Biomedicals, LLC, Orangeburg, NY, USA). The detection limit of the assay was 7.7 ng/mL.

### Immune cells phenotyping

#### Flow cytometry

To evaluate the phenotype of distinct immune system populations by flow cytometry, single-cell suspensions from spleen and thymus were prepared. Splenic erythrocytes were depleted by incubation with a hemolytic solution (155 mM NH_4_Cl, 10 mM KHCO_3_, pH 7.2) for 5 min at room temperature. For cell surface staining, 5 × 10^5^ cells were used from each individual rat and incubated with specific antibodies, according to Table 1, for 20 min on ice. Cell surface markers were analyzed using specific antibodies for CD11 b/c (OX-42), CD45RA (OX-33) (Caltag, CA, USA), CD161 (10/78), CD4 (W3/25), CD8 (G28) (BioLegend, San Diego, CA, USA), and CD3 (G4.18) (eBiosciences, San Diego, CA, USA). Cells were fixed with 2% formaldehyde after staining. Fifty thousand events were acquired on a FACSCalibur flow cytometer (Becton Dickinson, NJ, USA) using the Cell Quest software (Becton Dickinson, NJ, USA); analyses of the cell populations were performed using FlowJo software (Tree Star, Ashland, OR, USA).

**Table 1:** **Antibodies used for the identification of different immune cells**.

Immune cells	Phenotype spleen cells	
CD4^+^ T cells	CD3^+^ CD4^+^ (gated lymphocytes in FSC, SSC)	
CD8^+^ T cells	CD3^+^ CD8^+^ (gated lymphocytes in FSC, SSC)	
B cells	CD45Ra^+^ (gated lymphocytes in FSC, SSC)	
Macrophages	CD11bc^+^ (gated in all cells except granulocytes in FSC, SSC)	
Granulocytes	CD11bc^+^ granulocytes^+^ (gated granulocytes in FSC, SSC)	
NK cells	CD3^−^CD161high^+^	

	**Phenotype thymocytes**	

DN	CD4^−^CD8^−^	(gated lymphocytes in FSC, SSC)
DP	CD4^+^CD8^+^	
CD4 SP	CD4^+^CD8^−^	
CD8 SP	CD4^−^CD8^+^	

### *In vitro* thymocyte treatment with dexamethasone

Thymocytes were resuspended in DMEM (supplemented with 10% heat inactivated FCS, 10 mM HEPES buffer, 1 mM sodium pyruvate, 2 mM l-glutamine, 50 μg/mL streptomycin, and 50 U/mL penicillin, all from Invitrogen, CA, USA), plated into 96-well plates (1.5 × 10^6^ cells/mL), and treated, with or without 10 μM of dexamethasone (Sigma, St Louis, USA), for 4 h. To analyze cell death, cells were stained for CD4 (W3/25), CD8 (G28), annexin V, and 7AAD (BioLegend, San Diego, CA, USA), in accordance with Table 2 and the manufacture instructions. Cells were analyzed by flow cytometry as already described.

**Table 2:** **Identification of cell death**.

	Phenotype
Alive	Annexin V^−^7AAD^−^
Apoptosis	Annexin V^+^ 7AAD^−^
Necrosis	Annexin V^±^7AAD^+^

### Statistical analysis

To calculate the number of animals used in the experiments, we performed a power analysis (using G*Power 3.1.7). To compare the three independent groups, an one-way ANOVA test should be used and, assuming a large effect size (*f* = 0.4), an alpha of 0.05, and a statistical power of 0.5, 36 animals (12/group) were needed. In order to confirm our results, we performed two independent experiments. All dependent variables were assessed for normality. One-way ANOVA, followed by *post hoc* Bonferroni tests (when main effects were observed significant), was performed in order to assess group differences. To estimate the effect size, we calculated the η^2^ (dividing the between-groups sum of squares by the total sum of squares); η^2^ ≥ 0.01 indicates a small, ≥0.06 medium, and ≥0.14 large effects (Cohen, 1988).

A linear regression was conducted in order to predict immobility in the FST, using corticosterone levels and T cell CD4/CD8 ratio as potential predictors. Significance is referred as *p < 0.05.

## Results

### Animals submitted to MS_2–15_ displayed depressive-like behavior

**Figure 1:**
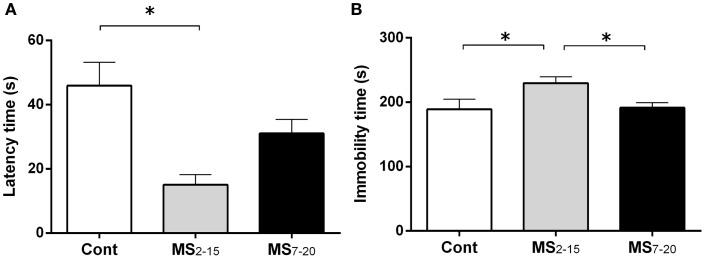
**Early maternal separation (MS_2–15_) induced depressive-like behavior**. **(A)** Latency to first immobility and **(B)** duration of immobility in the FST were assessed in Cont, MS_2–15_, and MS_7–20_ groups at 3 months of age. Each bar represents the mean + SEM from 11 to 20 rats per group from one of two independent experiments.

**Figure 2:**
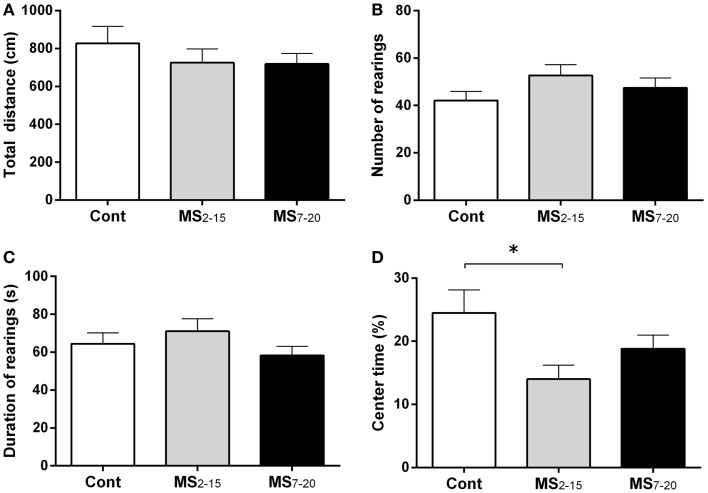
**MS_2–15_ group presented anxious-like behavior in the OF test**. The OF test was performed to assess **(A)** total distance, **(B)** number, **(C)** duration of rearings, and **(D)** time spent in the center of the OF arena in Cont, MS_2–15_, and MS_7–20_ groups at 3 months of age. Each bar represents the mean + SEM from 11 to 20 rats per group from one of two independent experiments.

In the FST, only the animals from the group separated earlier (MS_2–15_) displayed shorter latency time to immobility (Figure 1A; *F*_2,41_ = 7.92, *p* = 0.001 and η^2^ = 0.28) when compared to the Cont group (*p * = 0.009). Accordingly, MS_2–15_ displayed a significant increase in the immobility time (Figure 1B; *F*_2,41_ = 4.41, *p* = 0.02 and η^2^ = 0.18) when compared with Cont animals (*p * = 0.049) and with MS_7–20_ group, in the FST (*p* = 0.03). No significant differences were observed between MS_7–20_ and Cont group (Figure 1B; *p* = 0.997). Considering that FST is a highly demanding motor task, we performed the OF test to control for locomotor impairment that could underlie the significant reduction of activity observed in the FST. No differences were observed between groups in the total distance traveled in the arena (Figure 2A; *F*_2,42_ = 0.66, *p* = 0.52) and in the number (Figure 2B; *F*_2,42_ = 1.25, *p* = 0.30) and duration (Figure 2C; *F*_2,42_ = 1.4, *p* = 0.30) of rearings. In fact, MS, irrespectively from when it occurred, did not affect spontaneous locomotion (Figure 2A) or exploratory behavior (Figures 2B,C). Conversely, OF results also demonstrated that the percentage of time spent in the center of the arena was significantly reduced in the MS_2–15_ (Figure 2D; *F*_2,42_ = 3.41, *p* = 0.04, and η^2^ = 0.14), when compared to Cont animals (*p* = 0.04), which is a sign of anxious-like behavior. MS_7–20_ did not differ from both Cont (*p* = 0.41) and MS_2–15_ (*p* = 0.52) groups in the percentage of time spent in the center of the arena.

### Both periods of MS caused increased corticosterone levels but only early MS triggered thymic and splenic cell alterations

**Figure 3:**
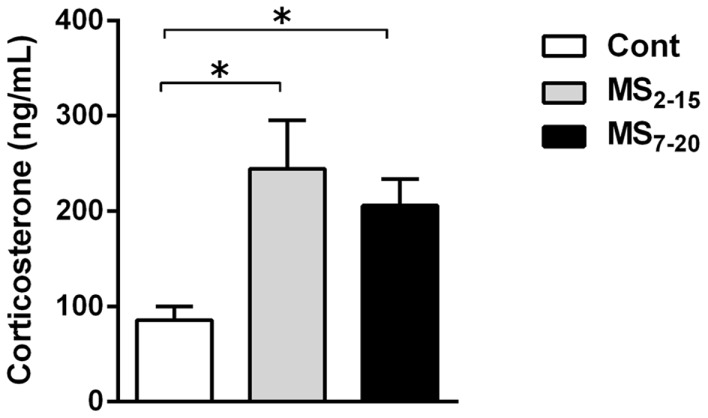
**Maternal separation induced increased corticosterone serum levels**. Each bar represents the mean + SEM from 16 to 21 rats per group from two independent experiments.

Corticosterone assessment revealed a long-lasting increase in basal corticosterone levels in both MS groups when compared with Cont animals (Figure 3; *F*_2,52_ = 6.58, *p* = 0.003, and η^2^ = 0.20; Cont vs. MS_2–15_ *p* = 0.003 and Cont vs. MS_7–20_ *p* = 0.04).

**Figure 4:**
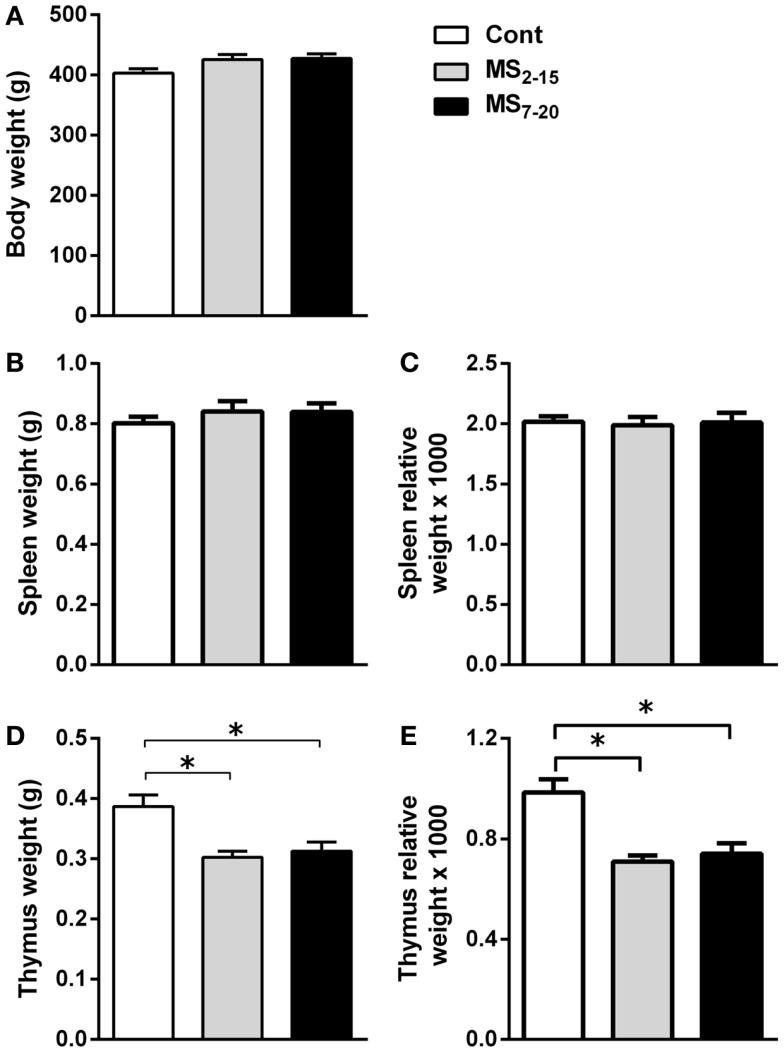
**Maternal separation caused a decrease in the thymus weight**. **(A)** Body weight, **(B)** spleen weight, **(C)** spleen relative weight, **(D)** thymus weight, and **(E)** thymus relative weight were measured in Cont, MS_2–15_, and MS_7–20_ groups at 3 months of age. Each bar represents the mean + SEM from 18 to 27 rats per group from two independent experiments.

**Figure 5:**
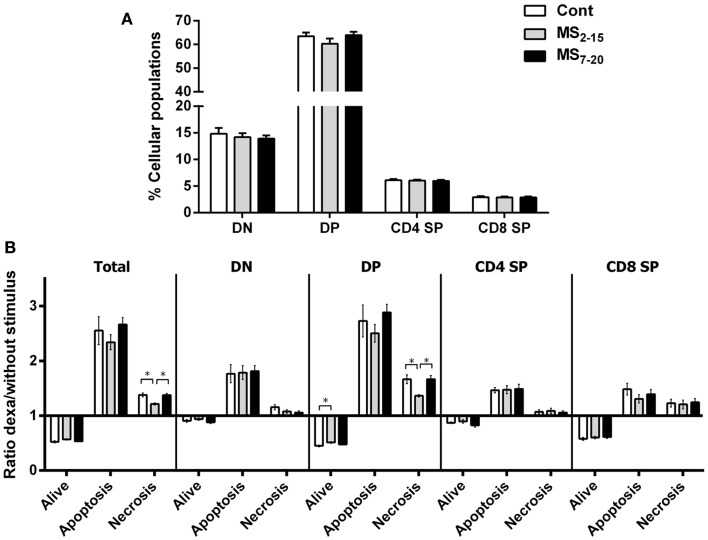
**Maternal separation did not alter the proportion of the main thymic populations but thymocytes from MS_2–15_ animals presented increased resistance to dexamethasone treatment**. **(A)** Thymocytes from Cont, MS_2–15_, and MS_7–20_ groups were stained with antibodies specific for D4 and CD8 and analyzed by flow cytometry. Each bar represents the mean + SEM from 19 to 26 rats per group from two independent experiments. **(B)** Thymocytes from Cont, MS_2–15_, and MS_7–20_ groups were treated during 4 h with dexamethasone followed by specific staining (Table 2) to evaluate apoptosis and necrosis of each cell population by flow cytometry. The data are represented as the ratio between the percentages of dexamethasone treated and untreated cells. Each bar represents the mean + SEM from 11 to 14 rats per group.

**Figure 6:**
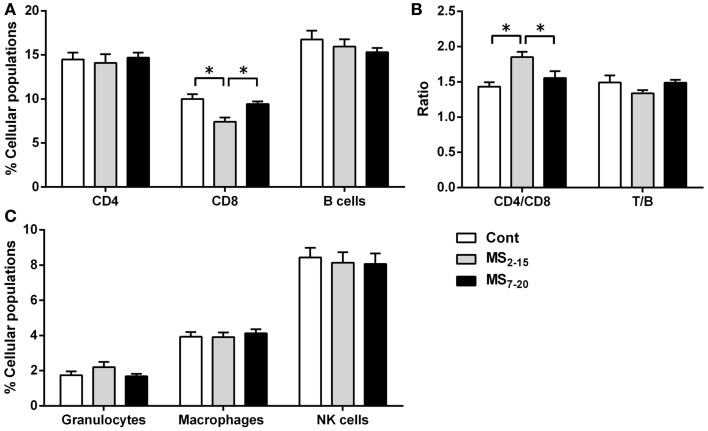
**Maternal separation between days 2 and 15 caused a decrease in the percentage of CD8^+^ T cells and an increase in the T cell CD4/CD8 ratio in the spleen of adult animals**. Splenocytes from Cont, MS_2–15_, and MS_7–20_ groups were labeled with specific antibodies (according to Table 1), and analyzed by flow cytometry, to identify the main spleen cell populations of the **(A)** adaptive immune system; **(B)** the ratios CD4/CD8 T and T/B cells; and **(C)** the innate cells of the immune system. Each bar represents the mean + SEM from 24 to 27 rats per group from two independent experiments.

Given the high sensitivity of the immune system to alterations in corticosteroid *millieu*, we analyzed the impact of MS on the weight of two of the most relevant immune system organs, the thymus, the primary lymphoid organ that supports T cell differentiation, and the spleen, a central organ of the peripheral immune system. No alterations were observed in the absolute (Figure 4B; *F*_2,69_ = 0.58, *p* = 0.56) and relative weight of the spleen (normalized to the body weight; Figure 4C; *F_2_*_,66_ = 0.05 and *p* = 0.95) between the three groups. However, MS animals showed a significant reduction in the thymus weight when compared with Cont (Figure 4D; *F*_2,64_ = 8.75, *p* = 0.0004, and η^2^ = 0.22; Cont vs. MS_2–15_ *p* = 0.001 and Cont vs. MS_7–20_ *p* = 0.003); these differences were independent of the absolute animal body weight, since the same alterations were present when the thymus weight was normalized to the body weight (Figure 4E; *F*_2,62_ = 12.62, *p* < 0.0001, and η^2^ = 0.28; Cont vs. MS_2–15_ *p* = 0.0001 and Cont vs. MS_7–20_ *p* = 0.0003). Moreover, no differences were seen on the body weight of those animals (Figure 4A; *F*_2,67_ = 2.80 and *p* = 0.07). Taking into account the decreased thymic weight, we next analyzed whether this was reflected in the proportion of the four main thymic cell populations: double negative (DN: CD4^−^CD8^−^), double positive (DP: CD4^+^CD8^+^), CD4 single positive (CD4SP: CD4^+^CD8^−^), and CD8 single positive (CD8SP: CD4^−^CD8^+^). No differences on thymic cell populations were present (Figure 5A; DN: *F*_2,63_ = 0.30, *p* = 0.74; DP: *F*_2,63_ = 1.25, *p* = 0.29; CD4SP: *F*_2,63_ = 0.06, *p* = 0.94; CD8SP: *F*_2,63_ = 0.01, *p* = 0.99). We further studied the resistance of those populations to *in vitro* exposure to dexamethasone. Thymocytes from the MS_2–15_ group presented a decreased necrosis rate when compared to Cont and MS_7–20_ (Figure 5B; *F*_2,34_ = 9.75, *p* = 0.0005, and η^2^ = 0.34; Cont vs. MS_2–15_ *p* = 0.002 and MS_2–15_ vs. MS_7–20_ *p* = 0.01) and these alterations seemed to be mainly caused by the decreased necrosis observed among DP cells (Figure 5B; *F*_2,35_ = 8.82, *p* = 0.0008, and η^2^ = 0.34; Cont vs. MS_2–15_ *p* = 0.004 and MS_2–15_ vs. MS_7–20_ *p* = 0.002). Moreover, thymus from MS_2–15_ animals also presented a lower proportion of alive DP cells when compared with Cont (Figure 5B; *F*_2,34_ = 5.11, *p* = 0.011, and η^2^ = 0.23; Cont vs. MS_2–15_ *p* = 0.011). Regarding the peripheral immune system, a decreased percentage of CD8^+^ T cell was observed in the spleen of MS_2–15_ group when compared with both Cont and MS_7–20_ (Figure 6A; *F*_2,68_ = 8.54, *p* = 0.0005, and η^2^ = 0.20). This led to an increased T cell CD4/CD8 ratio in the MS_2–15_ group (Figure 6B; *F*_2,64_ = 6.87, *p* = 0.002, and η^2^ = 0.18), while no alterations were seen in the T/B cells ratio. No differences were observed in the other spleen cell populations analyzed (Figures 6A,C; CD4^+^ T cells, B cells, granulocytes, macrophages, and NK cells).

### Sera corticosterone levels and T cell CD4/CD8 ratio predict depressive-like behavior

The use of a linear regression model to predict depressive-like behavior revealed that corticosterone, *per se*, was a marginal statistical significant predictor of immobility in the FST (*F*_1,29_ = 4.02, *p * = 0.054). Instead, when both variables: corticosterone levels and the T cell CD4/CD8 ratio were included, a significant model emerged (*F*_2,28_ = 9.71, *p * = 0.001), explaining 37% of the variance. Increased corticosterone levels and higher T cell CD4/CD8 ratio were significant predictors of increased immobility in the FST (Table 3).

**Table 3:** **Regression analysis predicting depressive-like behavior**.

	Variables	*R*^2^ (*R*^2^_adj_)	*F* (df)	β	*t*
Step 1	Corticosterone level	0.12(0.09)	4.02 (1, 29)^#^	0.35	2.0^#^
Step 2	Corticosterone level	0.41(0.37)	9.71 (2, 28)*	0.44	3.0**
	T cell CD4/CD8 ratio		0.54	3.7**

^#^*p* = 0.05; *p < 0.05; **p < 0.01.

## Discussion

This study addressed the long-term effects of two time windows of MS on the HPA axis, immunological function, and depressive/anxious-like behaviors. We observed that, irrespectively of the time window, MS caused increased corticosterone sera levels in adulthood. However, only animals submitted to MS between the 2^nd^ and the 15^th^ postnatal days displayed immunological and behavioral alterations. Additionally, corticosterone sera levels and the T cell CD4/CD8 ratio were shown to predict depressive-like behavior in this animal model.

The comparison between two time periods of MS sheds light on the critical periods of development in which early life stress strongly impacts on mood behavior, immune, and endocrine systems. The earlier separation period (MS_2–15_) mimics the temporal window most widely used in the literature and overlaps most of the stress hypo-responsive period (Schmidt et al., 2011), while the MS_7–20_, with the same exposure duration and some overlap, occurs when some components of the HPA axis regulation are known to be already mature (Schmidt et al., 2011). Remarkably, both MS exposure periods impacted similarly on the adult HPA axis basal function leading to increased basal corticosterone levels, suggesting that the postnatal week common to both MS periods (from postnatal day 7 to 15, last days of the stress hypo-responsive period) is determinant in the programing effects on the HPA axis. Since this time window is critical for the maturation of the HPA axis, it is not surprising that the stress triggered by MS performed during this period, caused a long-lasting disruption in the HPA axis function leading to increased basal levels of corticosterone in the adult progeny. Similar results were observed by several other authors, even using slightly distinct MS protocols (Clarke, 1993; Slotten et al., 2006; Batalha et al., 2013). Conversely, decreased (Slotten et al., 2006) or even no alteration (Plotsky et al., 2005) in corticosterone basal levels in animals submitted to MS were also reported. Overall, most literature seems to corroborate that MS is a developmental disruptor of the neuroendocrine function with long-lasting effects on the HPA axis activity and responsiveness. However, even MS animals that do not present alterations in the basal levels of corticosterone show a hypersecretion of this molecule in response to a psychological stressor (Plotsky et al., 2005).

Of notice, anxious and depressive-like behaviors in adulthood were found only when MS was applied earlier, which indicates the existence of sensitive periods for stress-related behavioral programing. It would be interesting to next evaluate other dimensions of depressive-like behavior. Even though the behavioral test used in this study (FST) is the one most widely used to assess behavior despair in rodents (Petit-Demouliere et al., 2005; Sousa et al., 2006), it also displays strong correlations with other dimensions of depression such as hedonic behavior (Bessa et al., 2009b). The impact on mood observed in the MS_2–15_ group is in accordance with previous studies performed in the same developmental window (Kalinichev et al., 2002; Ruedi-Bettschen et al., 2005; Lee et al., 2007; Lambas-Senas et al., 2009; Holsboer and Ising, 2010) while, to our knowledge, no other studies evaluated mood in animals submitted to MS_7–20_.

The observed stress consequences on adult corticosterone levels and behavior observed in the MS_2–15_ group are unlikely a consequence of altered maternal behavior, since in a previous study we observed no maternal behavior differences or altered corticosteroid levels in the dams, 2 weeks after delivery (Mesquita et al., 2007). However, the maternal behavior of the MS_7–20_ group was not assessed and, indeed, some reports stated an increased active maternal care in animals submitted to MS that seems to buffer potential consequences of long separation periods (Zhang et al., 2004; Macri et al., 2008). This could be a potential mechanism by which MS_7–20_ are different from MS_2–15_; further investigation will help to clarify this issue.

Interestingly, the differential behavioral alterations observed in MS groups show that factors other than the HPA axis are necessary for the induction of anxious and depressive-like behaviors. We searched for parameters of the immune system. Of notice, we observed thymic atrophy in both MS treated groups, which may likely result from the well-described effect of the glucocorticoids (similarly increased in both MS groups) on the thymus (Ashwell et al., 2000a). To our knowledge, this is the first study that describes a decreased adult thymic weight in animals submitted to MS, associated with higher levels of corticosterone. Chen et al. (2012) have also analyzed male rats submitted to 4 h MS during the 2^nd^ and the 13^th^ postnatal day and failed to observe alterations in the thymic weight, as well as any alterations in basal levels of corticosterone. Curiously decreased thymic weight and increased serum levels of corticosterone are common alterations in both MS groups. Since the two MS periods used in this study overlap for 1 week, this suggests that the postnatal day 7–15 seems crucial for the programing effect in the corticosterone levels and that it may lead to the alterations in the thymus weight and in number of cells observed. Of interest, despite the decrease in thymus size, no alteration in the proportion of the four main thymocyte populations was observed. These results are in accordance with those from Kruschinski et al. (2008) in which MS was performed from postnatal day 1 to 28. However, in the present report, after *in vitro* dexamethasone treatment (known to induce thymocyte death) only the MS_2–15_ group presented increased resistance to cell death; mainly due to the increased resistance in the DP cells, the thymocyte population know to be more susceptible to glucocorticoids-induced cell death (Ashwell et al., 2000b). The differential response of the MS groups indicates that additional programing alterations exist in the thymocytes causing glucocorticoid resistance when MS is imposed earlier (MS_2–15_). One of the mechanisms that may underlie the alterations in the thymocytes resistance in the MS_2–15_ group is an impaired glucocorticoid receptor (GR) function of the thymocytes. Such dysfunction in GR has also been associated with alterations in the cytokine milieu (Silverman and Sternberg, 2012). We did not analyze the cytokine profile of these animals, but alterations in the cytokine milieu of animals submitted to MS during the first 2 weeks of life have been shown by others (Dimatelis et al., 2012; Avitsur et al., 2013), which can be indicative of a simultaneous mechanism that contributes to the thymocyte glucocorticoid resistance observed in the MS_2–15_ group.

The effect of MS on the peripheral immune system also seems to be time specific. Only the earlier period of MS caused a decrease in the percentage of CD8^+^ T cells in the spleen and, consequently, an increase in the T cell CD4/CD8 ratio. Further supporting that timing of MS is crucial when MS is imposed to rats between postnatal days 1 and 28, Kruschinski et al. (2008) did not observe alterations in spleen immune cell populations. The T cell alterations observed in the spleen of MS_2–15_ animals do not seem to be a consequence of impairment in thymic T cell differentiation, since no differences in the four main population of the thymus were observed; but rather an alteration in the peripheral homeostatic mechanisms (Rocha et al., 1989). The decreased percentage of CD8^+^ T cells in the spleen can be caused by a deficient signaling to support cell survival or to an increased cell death in these cells, which deserve further investigation. Interestingly, a recent study showed that CD8^+^ T cells display a more pronounced expression of dopaminergic transporters and receptors when compared with CD4^+^ T cells (Mignini et al., 2013) and, dopamine was shown to inhibit the proliferation of T cells, particularly of CD8^+^ T cells (Saha et al., 2001). Given that MS impacts on the dopaminergic system, it is plausible that this might constitute an additional mechanism for the present finding of reduced CD8^+^ T cells (Li et al., 2013). Moreover, these T cells are primarily involved in immune response to pathogens (mainly virus) and tumor cells, and are also implicated in transplant rejection. Curiously, C56BL/6 mice exposed to MS between postnatal days 1 and 14 were shown to present increased susceptibility to influenza virus infection; however, the levels of CD8^+^ T cells were not evaluated in these study (Avitsur et al., 2006).

Our findings are novel in revealing an increased T cell CD4/CD8 ratio, which is a highly preserved ratio within strains of animals (Rocha et al., 1989; Sim et al., 1998). Of notice, another rodent model of depressive-like behavior, induced by prenatal administration of dexamethasone, showed that adult males similarly display increased T cell CD4/CD8 ratio and that the percentage of CD8^+^ T cells is negatively correlated with the latency in the FST (Roque et al., 2011). In humans and monkeys submitted to early life stress, alterations in T cell CD4/CD8 ratio have also been reported (Lewis et al., 2000; Gogberashvili, 2006), even though in the opposite direction of what we describe herein. Remarkably, our results reveal that differences in immune cell populations are only present in animals displaying behavioral alterations, suggesting that the programing effects caused by MS impact not only in the endocrine system but also in the immune and central nervous systems, highlighting the interplay between them. Curiously, lower proportion of CD8^+^ T cells and a higher T cell CD4/CD8 ratio have been associated with lower levels of hippocampal neuronal proliferation (Huang et al., 2010). Moreover, although disputable, most studies show that MS decreases hippocampal cell proliferation (Hulshof et al., 2011), which in itself is associated with depressive-like behavior (Bessa et al., 2009a; Song and Wang, 2011).

The fact that the two MS periods do not overlap in the first and third postnatal week could also be crucial for the distinct effects, we observed in behavior and in parameters of the immune system. Considering that during the first postnatal week significant differences in the number and maturation process of GR in the hypothalamus, pituitary, and hippocampus were observed (De Kloet et al., 1988), one can speculate that stressful events during this very immature period could be fairly impactful on long-term behavior. In fact, early life events may lead to long-term epigenetic effects such as down regulation of hippocampal GR through the increased methylation patter in the coding gene (Weaver et al., 2004). A similar mechanism could also be caused by very early MS, since the first postnatal week is the most immature period of the HPA axis control. In the MS_7–20_ period, the HPA axis already presents an increased degree of maturation and number of GR in critical regions for HPA axis control (Vazquez and Akil, 1993). Of interest, GR are solely found in the hippocampus in the second postnatal week (Vazquez and Akil, 1993). The presence of GR (even in low levels) during the second and third postnatal week could be crucial for the organism to respond to stressful situations and buffer some of the long-term behavioral effects, such as anxious and depressive behaviors. Although speculative, this could be a likely explanation since we observed an increased anxious and depressive-like phenotype in the earlier MS group (MS_2–15_) and not in the later one (MS_7–20_).

As an attempt to assess the role of both corticosterone levels and T cell CD4/CD8 ratio in the depressive-like behavior observed in our MS model, we performed a regression analysis. Both predictors explained 37% of the immobility time in the FST, suggesting a synergetic contribution of endocrine and immunological factors in the prediction of the depressive-like behavior observed in the MS animals. Of notice, corticosterone *per se*, accounted only for 9% of the variance and was merely a marginal significant predictor. We failed to find any predictors of anxious-like behavior. One should take into account that the depressive-like behavior, the increased corticosterone levels, and the alterations in the immune system seem to be related and caused by the effect of MS in an early life period. Even though additional studies are needed, our data suggests that this information might be potentially used to determine whether the predictive model for the depressive-like behavior can be a tool to predict if children submitted to adverse experiences in early life are more prone to develop mood disorders, which could eventually help in anticipating diagnosis of depression.

Altogether, this study shows that the timing in which early life stress is applied determines its long-lasting effects in several body systems, and that these interact in ways that may be predictive of mood behavior alterations in the adulthood.

## Author Contributions

S. Roque performed the immune cells phenotyping experiments and analysis. A. R. Mesquita performed the MS protocol, the behavior assessment and analysis, and the corticosterone measurement. Both S. Roque and A. R. Mesquita performed the statistical data analysis. All authors contributed to the planning of the experiments, data interpretation, writing of the manuscript, and approved the final version of the manuscript.

## Conflict of Interest Statement

The authors declare that the research was conducted in the absence of any commercial or financial relationships that could be construed as a potential conflict of interest.
